# Causes and consequences of the opioid epidemic in the Netherlands: a population-based cohort study

**DOI:** 10.1038/s41598-020-72084-6

**Published:** 2020-09-17

**Authors:** Ajda Bedene, Eveline L. A. van Dorp, Tariq Faquih, Suzanna C. Cannegieter, Dennis O. Mook-Kanamori, Marieke Niesters, Monique van Velzen, Maaike G. J. Gademan, Frits R. Rosendaal, Marcel L. Bouvy, Albert Dahan, Willem M. Lijfering

**Affiliations:** 1 Department of Clinical Epidemiology, Leiden University Medical Center, Albinusdreef 2, 2333 ZA Leiden, The Netherlands; 2 Department of Anesthesiology, Leiden University Medical Center, Leiden, The Netherlands; 3 Department of Internal Medicine, Section of Thrombosis and Hemostasis, Leiden University Medical Center, Leiden, The Netherlands; 4 Department of Primary Care, Leiden University Medical Center, Leiden, The Netherlands; 5 Department of Orthopedics, Leiden University Medical Center, Leiden, The Netherlands; 6 Division of Pharmacoepidemiology and Clinical Pharmacology, Utrecht Institute for Pharmaceutical Sciences, University Utrecht, Utrecht, The Netherlands

**Keywords:** Health policy, Public health, Epidemiology, Outcomes research, Addiction

## Abstract

Over the past decade opioid use has risen globally. The causes and consequences of this increase, especially in Europe, are poorly understood. We conducted a population-based cohort study using national statistics on analgesics prescriptions, opioid poisoning hospital admissions and deaths in the Netherlands from 2013 to 2017. Pain prevalence and severity was determined by using results of 2014–2017 Health Interview Surveys. Between 2013 and 2017 the proportion of residents receiving opioid prescription rose from 4.9% to 6.0%, and the proportion of those receiving NSAIDs decreased from 15.5% to 13.7%. Self-reported pain prevalence and severity remained constant, as 44.7% of 5,119 respondents reported no pain-impeded activities-of-daily-living in 2014 (aRR, 1.00 [95% CI, 0.95–1.06] in 2017 vs 2014). Over the observation period, the incidence of opioid poisoning hospitalization and death increased from 8.6 to 12.9 per 100,000 inhabitants. The incidence of severe outcomes related to opioid use increased, as 3.9% of 1,343 hospitalized for opioid poisoning died in 2013 and 4.6% of 2,055 in 2017. We demonstrated that NSAIDs prescription decreased and opioid prescription increased in the Netherlands since 2013, without an increase in pain prevalence and severity. Consequently, the incidence of severe outcomes related to opioids increased.

## Introduction

Opioid prescription and associated medical complications have increased over the recent years, particularly in the United States but also in Europe^1, 2^. Around 800,000 individuals in the Netherlands received an opioid prescription in 2013, which increased to over one million individuals (6.0% of the total population) in 2017. Hospital admissions for opioid poisoning increased from 9.2 per 100,000 inhabitants in 2013 to 13.1 per 100,000 inhabitants in 2017, and opioid-related mortality increased 50% over the same four-year period^2^.

Causes for the increased frequency of opioid prescription have not been studied in Europe, although different explanations have been proposed^2^. The first cause could be an increased demand for analgesic prescription due to increasing pain prevalence in the population, possibly due to ageing and concomitantly increased morbidity^3, 4^. Nationwide data from the United States have indeed shown that people after the mid-1990s gradually reported increased frequency of pain^5^. A second reason could be a shift from nonsteroidal anti-inflammatory drugs (NSAIDs) to opioids prescription. Recently an increasing number of scientific publications have raised awareness that NSAIDs users are at increased risk for adverse events and interactions with other pharmaceutical agents^6, 7^. Simultaneously, the Dutch pain guidelines reintroduced oxycodone in analgesic clinical practice^8^. Indeed, preliminary data from the Netherlands showed a peak of NSAIDs use in 2011–2012, followed by a decrease, while concurrently an increase in oxycodone use has been noted^9^. However, it is unknown whether the increase in opioid prescription is paralleled by a similar decrease in NSAIDs prescriptions, which could offer an explanation of the opioid crisis.

Together with the increased opioid prescription, an increase in opioid related fatalities, hospitalization and death due to opioid poisoning, was observed. However, information whether hospitalization and death associated with opioid use were also boosted by an increase in illicit opioid use is currently unknown for the Netherlands. Studies from the United States have shown that wide-spread use of prescription opioids in the community preceded illegal opioid trade^10, 11^. The latter introduces an additional risk for severe outcomes, such as overdose, as users of illicit opioids are not monitored^12^. Therefore we assessed, among those who were hospitalized or died due to opioid poisoning, how many had not been reimbursed for an opioid prescription, which indicates either in-hospital administration or illicit use. Furthermore, we examined different outcomes of opioid poisoning, i.e., death, prolonged hospitalization or complete recovery, in order to estimate whether the opioid crisis deepened since 2013.

We aimed to explore two possible causes for increased opioid prescription in the Netherlands: increased pain prevalence and severity, and decreased NSAIDs prescription. Furthermore, we estimated consequences of increased opioid prescription such as hospital admission and death due to opioid poisoning on a population level. The causes and consequences of increased opioid prescription warrant knowledge on precautions needed to be made to prevent further increase in opioid-related fatalities.

## Methods

### Participants

We conducted a population-based cohort study in the Netherlands. Detailed method descriptions have previously been reported^2^. In brief, we performed analyses into prescription reimbursement data, hospitalization, and mortality data using several anonymized databases from Statistics Netherlands (CBS) covering the total population of the Netherlands between 2013 and 2017^13–16^. Furthermore, we used data from the national Health Interview Surveys, “Gezondheidsenquête” (GE) from the years 2014–2017 (also collected by the CBS)^17^. Datasets were linked on an individual level, based on the unique anonymized identifiers.

The institutional review board of the Anesthesiology Department and Intensive Care Unit of the Leiden University Medical Center approved the study in CBS and waived participant consent.

### Pain prevalence and severity

Information on pain perception was included in the GE surveys from 2014 to 2017. The GE is an annual national survey that covers health-related lifestyle choices of Dutch residents^17^. Since 2013, the GE surveys have been conducted as part of the European Health Interview Survey (EHIS) and direct comparisons between the yearly GEs can be made for the years 2014–2017^18, 19^. The survey is sent to a random subset of the population of around 15,000 individuals each year, with response rates between 60 and 65%^17^. Over the four-year period around 38,000 individuals participated in the survey.

To explore the prevalence and severity of pain in the population, we investigated pain-impeded activities of daily living (ADL). Pain-impeded ADL was defined as the level of performance of daily activities (including outdoor and household chores) hindered by pain in the past four weeks^17^. Pain-impeded ADL was asked on a five-point Likert scale, with the categories: “not at all”, “somewhat”, “moderate”, “much”, and “extreme”. Due to a low number of respondents in the “much”, and “extreme” categories, these two groups were merged into the category “much and extreme”.

### Analgesic prescription

Prescription reimbursement data were collected for all Dutch residents entitled to pharmaceutical care, i.e., those with health insurance, which is 99.9% of all Dutch residents^20^. The Dutch Health Care Institute (ZINL) provides these data to the CBS. Analgesics dispensed from outpatient, community pharmacies, and in residential homes for elderly are collected in the national reimbursement database, whereas medicines dispensed in hospitals and nursing homes are not^14^.

Opioid prescriptions were classified according to the ATC (the WHO drug classification system) code N02A and further stratified by natural (N02AA) and synthetic (N02AZ) opioids^21^. Morphine (N02AA01), hydromorphone (N02AA03), nicomorphine (N02AA04), and oxycodone (N02AA05) were classified as natural opioid (N02AA), and pethidine (N02AB02), fentanyl (N02AB03), dextromoramide (N02AC01), piritramide (N02AC03), pentazocine (N02AD01), buprenorphine (N02AE01), codeine with paracetamol (N02AJ06), tramadol with paracetamol (N02AJ13), tramadol (N02AX02), tapentadol (N02AX06), and other opioids (N02AX52) were classified as synthetic opioid (N02AZ)^22^. NSAIDs were classified according to ATC code M01A^21^. Individuals were considered exposed to prescription drugs when they filled at least one prescription per studied calendar year.

### Hospital admissions and deaths related to opioid use

The Dutch Hospital Database contains information about all-cause hospital admissions and the Register of Causes of Death records all-causes of death. Each hospital admission record contains the date of hospital inpatient and outpatient encounters, the discharge date, and the discharge diagnoses^15, 16^. Diagnoses and deaths are coded within the CBS according to the International Statistical Classification of Diseases and Related Health Problems (ICD, 10th revision) of the WHO^23^.

Hospital admissions and deaths registered as due to opioid-related disorders, adverse events of opioid use and opioid overdose were defined as opioid poisoning (see Supplement online)^24^. Opioid poisoning cases were selected based on first hospital admission or death, whichever occurred first, per studied calendar year.

### Statistical analysis

We present descriptive statistics for all Dutch residents between 2013 and 2017 on opioid prescriptions, hospital admissions and mortality. Individuals, who received opioid or NSAIDs prescriptions are presented as counts and as a proportion of the total population per calendar year. Hospital admissions, deaths, and also prescriptions are presented as number per 100,000 inhabitants per calendar year and as risk ratios with 95% confidence interval (CI) compared with the reference year (2013). Risk ratios were adjusted for age and sex (aRR) with direct standardization; age was divided in 5 categories (0–15, 15–25, 25–45, 45–65, ≥ 65 years), and further stratified by sex with weights from the total Dutch population of 2013. Similar analyses were performed on the 2014–2017 GE cohorts where the selected reference group was the 2014 GE cohort, whence the weights for standardization analysis were selected. To investigate whether GE surveys were a valid representation of the total population, we linked GEs data with the prescription datafiles of the same calendar year, and performed frequency analysis into opioid and NSAIDs prescription among respondents of the GEs.

#### Missing data

Individuals with missing data in the pain-impeded ADL variable in the GEs were dropped from the analysis (n = 4,569, 46.5% in 2017). In 2013, 8 (0.6%) hospitalization cases due to opioid poisoning had missing information about residence before and destination after hospitalization. In 2014, 31 (2.2%) cases had missing information about residence before hospitalization due to opioid poisoning, and 29 (2.0%) cases had missing information about destination after hospital admission due to opioid poisoning. After 2014, there were no missing data. There were no missing data for the total population characteristics and no individuals were lost in the linkage process.

#### Hospital admissions and deaths related to opioid use

To study the impact of increased opioid prescription we performed several analyses. First, we estimated opioid poisoning per calendar year (either leading to hospitalization or death). Then we stratified opioid poisoning cases in two categories: whether individuals received opioid prescription or none in the same calendar year. This provides insight in severe outcomes—defined as death, or consequent transfer to another health facility after being hospitalized for opioid poisoning—related to prescription opioid use versus in-hospital or illicit opioid use. Second, we explored residence status prior to hospitalization. Those who were transferred from another health facility to a hospital for an opioid poisoning were considered poisoned whilst being hospitalized, which provides information about in-hospital opioid use. Third, we assessed the severity of opioid poisoning per calendar year by following-up patients after their hospitalization. We classified three main outcomes of opioid poisoning: returning home, prolonged institutionalization, or death. Those who were able to return to their own living environment were considered to have experienced a milder form of poisoning. Those who were transferred to another health facility were considered prolongedly institutionalized due to a more severe poisoning. Individuals who were transferred to a psychiatric hospital were considered having an opioid addiction. Patients who died after being hospitalized for opioid poisoning were considered having experienced an opioid overdose.

The STROBE statement checklist for cohort studies is included in the Supplement online. All statistical analyses were performed with SPSS for Windows, release 24.0 (SPSS, Chicago, IL, USA). Figures were created with R studio (A Language and Environment for Statistical Computing, R Core Team, R Foundation for Statistical Computing, Vienna, Austria, https://www.R-project.org), using *R* package *ggplot2* version 3.2.1^25^.

## Results

### Description of the studied populations

For the evaluation of analgesic prescription practice and consequences of changes thereof we studied 2 populations: the total Dutch population between 2013 and 2017, and the GE survey participants between 2014 and 2017.

For the total Dutch population, among the 16,779,575 (mean age, 40.8 years) residents in 2013, 8,472,236 (50.5%) were women. In 2017, 8,606,405 (50.4%) were women for a total population of 17,081,507 (mean age, 41.6 years) (see Supplementary Table S1 online).

In the GE cohorts, 9,516 GE respondents were included in 2014 (mean [SD] age, 40.4 [23.7] years, 4,879 women, 51.3%). In 2017, 9,826 GE respondents were included (mean [SD] age, 41.7 [24.1] years, 4,978 women, 50.7%) (see Supplementary Table S1 online).

### Is there an increase in pain prevalence and severity?

The missing proportion for pain-impeded ADL in GE cohorts was approximately 46% and the level of missingness did not change between 2014 and 2017 (Table 1). Most of the respondents of the 2014–2017 GE surveys reported “not at all” (44.7% in 2014 and 44.3% in 2017) difficulties with pain-impeded ADL. Approximately the same proportion of the respondents of the 2014–2017 GE surveys had “moderate” (10.1% in 2014 and 9.6% in 2017) and “much and extreme” (9.0% in 2014 and 9.5% in 2017) levels of difficulty with ADL due to pain (Table 1). The age and sex-adjusted risk ratio (aRR) for “not at all” category in 2017 versus 2014 was 1.00; [95% confidence interval (CI), 0.95–1.06], and for “much and extreme” was 1.04; [95% CI, 0.91–1.18] (Table 1).

**Table 1:** Pain-impeded activities of daily living among the respondents of GE surveys, from 2014 to 2017.

		2014 (n = 9516)	2015 (n = 9358)	2016 (n = 9165)	2017 (n = 9826)
**Pain impeded activities of daily living**					
Not at all	No./Total No. (%)	2287/5119 (44.7)	2191/5021 (43.6)	2117/4947 (42.8)	2330/5257 (44.3)
	aRR (95% CI)*	1 (reference)	0.97 (0.92–1.03)	0.96 (0.90–1.02)	1.00 (0.95–1.06)
Somewhat	No./Total No. (%)	1857/5119 (36.3)	1861/5021 (37.1)	1819/4947 (36.8)	1923/5257 (36.6)
	aRR (95% CI)*	1 (reference)	1.02 (0.96–1.09)	1.01 (0.95–1.08)	1.01 (0.94–1.07)
Moderate	No./Total No. (%)	516/5119 (10.1)	502/5021 (10.0)	511/4947 (10.3)	506/5257 (9.6)
	aRR (95% CI)*	1 (reference)	1.00 (0.88–1.13)	1.02 (0.91–1.16)	0.94 (0.83–1.06)
Much and extreme	No./Total No. (%)	459/5119 (9.0)	467/5021 (9.3)	500/4947 (10.1)	498/5257 (9.5)
	aRR (95% CI)*	1 (reference)	1.04 (0.91–1.18)	1.12 (0.99–1.28)	1.04 (0.91–1.18)
Missing	No. (%)	4397 (46.2)	4337 (46.3)	4218 (46.0)	4569 (46.5)
	aRR (95% CI)*	1 (reference)	1.01 (0.97–1.06)	1.01 (0.97–1.05)	1.02 (0.98–1.06)

aRR, adjusted risk ratio; CI, confidence interval; GE, Health Interview Survey; NSAIDs, nonsteroidal anti-inflammatory drugs.

*Adjusted for age and sex with direct standardization; 2014 cohort was selected as a reference population in the GE survey.

### Changed analgesic prescription practice

**Figure 1:**
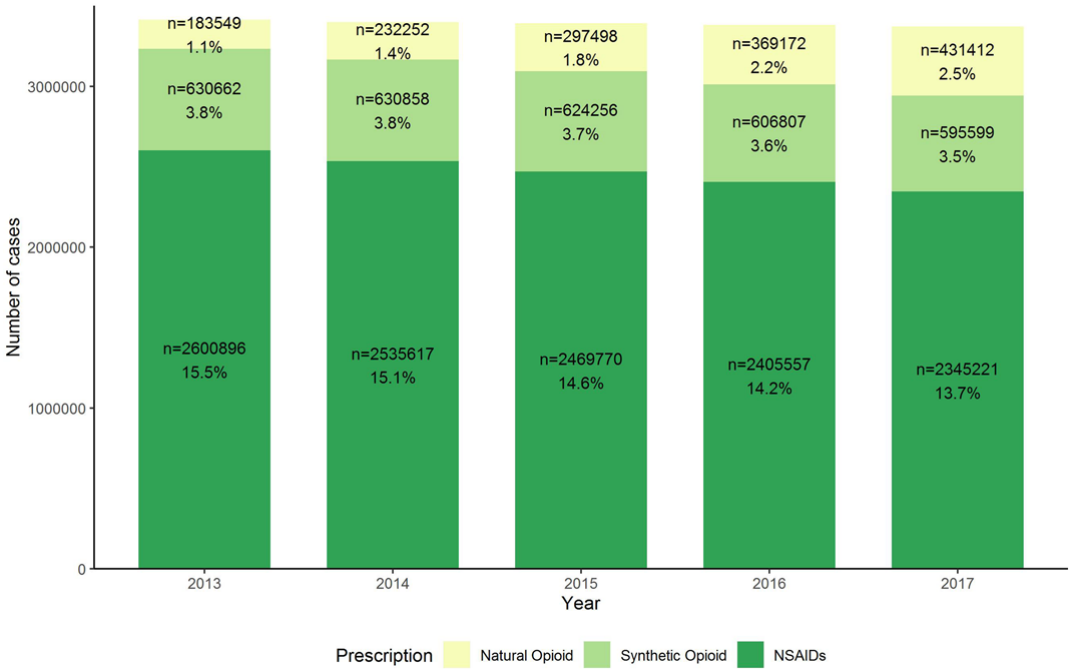
Opioid (overall and stratified by natural and synthetic), and NSAIDs prescription cases in the Netherlands, from 2013 to 2017. Abbreviations: NSAIDs, nonsteroidal anti-inflammatory drugs. Individuals, who reimbursed opioid prescriptions were selected by ATC code N02A (natural, N02AA; synthetic N02AZ), NSAIDs prescriptions by ATC code M01A.

Between 2013 and 2017 there was a 20% increase in opioid prescriptions (814,211, 4.9% in 2013; 1,027,019, 6.0% in 2017; aRR, 1.20; [95% CI, 1.20–1.20]) (Fig. 1 and Supplementary Table S2 online). Stratified analysis showed that natural opioids contributed most to that increase since their use more than doubled (1.1% in 2013, and 2.5% in 2017, aRR, 2.23; [95% CI, 2.22–2.24], whereas prescriptions of synthetic opioids prescription decreased slightly (3.8% in 2013, and 3.5% in 2017, aRR, 0.90; [95% CI, 0.90–0.90]). Between 2013 and 2017 the proportion of individuals who received NSAIDs prescriptions decreased (15.5% in 2013, and 13.7% in 2017, aRR, 0.88; [95% CI, 0.88–0.88]).

**Figure 2:**
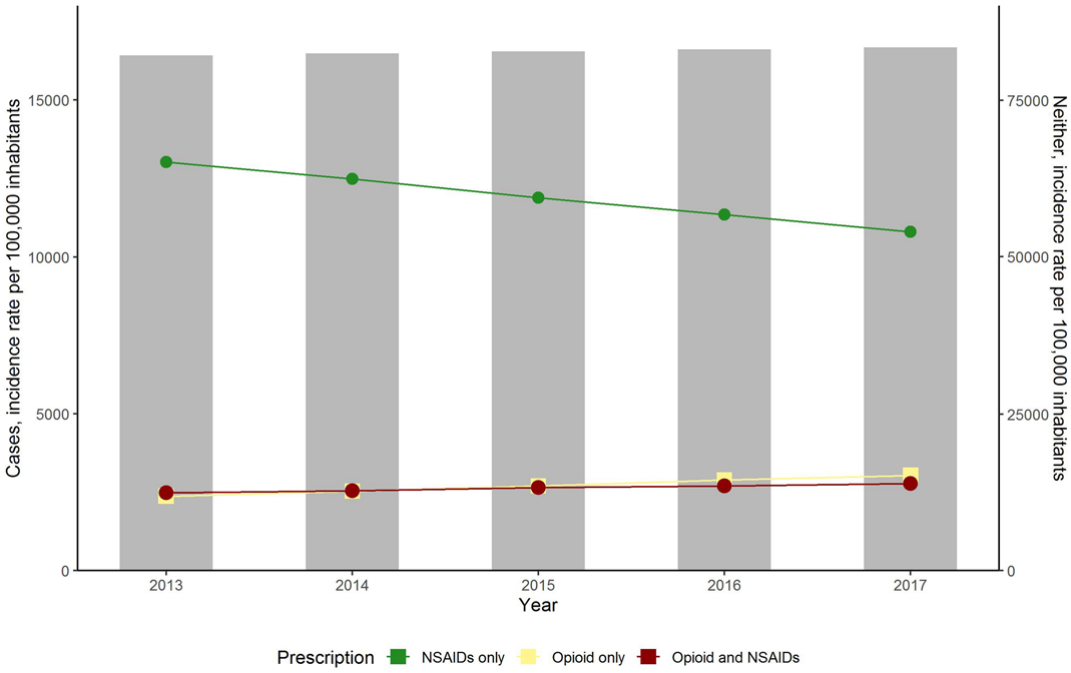
Opioid and NSAIDs prescription cases (stratified by concomitant and single prescription) and those with neither of these analgesic prescriptions in the Netherlands, from 2013 to 2017. Abbreviations: NSAIDs, nonsteroidal anti-inflammatory drugs. Individuals, who reimbursed opioid prescriptions were selected by ATC code N02A (natural, N02AA; synthetic N02AZ), NSAIDs prescriptions by ATC code M01A. Prescription cases are presented as incidence rates per 100,000 inhabitants per observed calendar year. Primary axis presents incidence of analgesics prescription, and the secondary axis shows the incidence of those with neither of analgesics prescription.

The number of individuals who only received an opioid prescription as analgesic increased with nearly 30% between the 2013 and 2017 (2.4% in 2013 and 3.2% in 2017; aRR, 1.28, [95% CI, 1.27–1.29]), the number of those who received an opioid and NSAIDs prescription increased slightly (2.5% in 2013 and 2.8% in 2017; aRR, 1.12 [95% CI, 1.12–1.13]), whereas the number of individuals who only received NSAIDs prescription decreased (13.0% in 2013 and 10.9% in 2017; aRR, 0.83 [95% CI, 0.83–0.83]) (Fig. 2 and Supplementary Table S3 online). The number of individuals with neither of these analgesic prescriptions remained stable from 2013 to 2017 (aRR, 1.02; [95% CI, 1.01–1.02], reference year 2013).

### Impact of increased opioid prescription on opioid poisoning

The frequency of opioid poisoning increased with nearly 50% between 2013 and 2017 (n = 1,440, 8.6 per 100,000 inhabitants in 2013, and n = 2,200, 12.9 per 100,000 inhabitants in 2017; aRR, 1.48; [95% CI, 1.39–1.59]) (Table 2). The frequency of opioid poisoning related to opioid prescriptions, nearly doubled after 2013 (3.4 and 6.6 per 100,000 inhabitants in 2013 and 2017, respectively; aRR, 1.89; [95% CI, 1.71–2.09]). The number of individuals who were hospitalized or died because of opioid poisoning, but had not filled a prescription at a pharmacy also increased, indicative of increase in illicit use (5.2 and 6.3 per 100,000 inhabitants in 2013 and 2017, respectively; aRR, 1.22; [95% CI, 1.11–1.33]). From 2016, the frequency of opioid poisoning among those who had received prescription opioids was equal/slightly surpassed that of those without a prescription (6.0 and 6.1 per 100,000 inhabitants, respectively in 2016 and 6.6 and 6.3 per 100,000 inhabitants, respectively in 2017). Patients with opioid poisoning without an opioid prescription were younger and more often male (mean [SD] age, 45.6 [17.8] years; n = 711 (66.3%) men) than poisoning cases related to opioid prescription (mean [SD] age, 56.4 [17.6] years; n = 514 (45.6%) men).

**Table 2:** Hospitalization and death of opioid poisoning, stratified by receiving opioid prescription in the Netherlands, from 2013 to 2017.

	2013 (n = 16,779,575)	2014 (n = 16,829,290)	2015 (n = 16,900,726)	2016 (n = 16,979,120)	2017 (n = 17,081,507)
**Opioid poisoning**					
No. (per 100,000)	1440 (8.6)	1499 (8.9)	1742 (10.3)	2049 (12.1)	2200 (12.9)
aRR (95% CI)*	1 (reference)	1.03 (0.96–1.11)	1.19 (1.11–1.28)	1.39 (1.30–1.49)	1.48 (1.39–1.59)
**Opioid prescription**					
No. (per 100,000)	572 (3.4)	696 (4.1)	844 (5.0)	1018 (6.0)	1127 (6.6)
aRR (95% CI)*	1 (reference)	1.20 (1.08–1.35)	1.44 (1.30–1.60)	1.73 (1.56–1.92)	1.89 (1.71–2.09)
Mean age (SD), years	56.1 (15.9)	57.8 (16.6)	57.3 (16.8)	55.9 (16.9)	56.4 (17.6)
Male, No. (%)	289 (50.5)	337 (48.4)	411 (48.7)	467 (45.9)	514 (45.6)
Female, No. (%)	283 (49.5)	359 (51.6)	433 (51.3)	551 (54.1)	613 (54.4)
**In-hospital and illegal opioid use**					
No. (per 100,000)	868 (5.2)	803 (4.8)	898 (5.3)	1031 (6.1)	1073 (6.3)
aRR (95% CI)*	1 (reference)	0.92 (0.84–1.02)	1.03 (0.94–1.13)	1.17 (1.07–1.28)	1.22 (1.11–1.33)
Mean age (SD), years	46.6 (16.4)	46.9 (16.2)	46.9 (16.9)	46.9 (18.1)	45.6 (17.8)
Male, No. (%)	564 (65.0)	534 (66.5)	583 (64.9)	658 (63.8)	711 (66.3)
Female, No. (%)	304 (35.0)	269 (33.5)	315 (35.1)	373 (36.2)	362 (33.7)

aRR, adjusted risk ratio; CI, confidence interval.

*Adjusted for age and sex with direct standardization; the 2013 total Dutch population cohort was selected as a reference.

Opioid poisoning cases were derived from hospitalization and death dataset for the year in concern, and duplicate cases were filtered out. ICD-10CM codes used to identify opioid poisoning are reported in the Supplement online. Opioid prescriptions were identified by ATC code N02A.

In 2017, most of the opioid poisoning cases (n = 1,943 out of total of 2,055; 94.5%) experienced disease onset outside of hospital, and 80 (3.9%) individuals experienced it whilst being hospitalized (Table 3). In 2013, the majority of individuals with opioid poisoning (1,085/1,343; 80.8%) were discharged home. That proportion decreased to 74.1% (1,523/2,055) in 2017 (RR, 0.92; [95% CI, 0.85–0.99]). However, an increasing number of patients were transferred from a hospital to another care facility after an opioid poisoning (205/1,343 (15.3%) in 2013, and 438/2,055 (21.3%) in 2017; RR, 1.40; [95% CI, 1.18–1.65]). Among these, transfers to a psychiatric hospital increased most in relative numbers (n = 41 (3.1%) patients in 2013, and n = 129 patients (6.3%) in 2017; RR, 2.06; [95% CI, 1.45–2.92]). Fatalities due to opioid poisoning also increased substantially, both in absolute numbers, and as a proportion of those with opioid poisoning, indicating an increase in severity of these cases (53/1,343 cases (3.9%) in 2013, and 94/2,055 cases (4.6%) in 2017; RR, 1.16; [95% CI, 0.83–1.62]).

**Table 3:** Residence before and destination after hospitalization for opioid poisoning in the Netherlands, from 2013 to 2017.

		**2013 (n = 16,779,575)**	**2014 (n = 16,829,290)**	**2015 (n = 16,900,726)**	**2016 (n = 16,979,120)**	**2017 (n = 17,081,507)**
**Opioid poisoning (hospital cases)**	No. (per 100,000)	1351 (8.1)	1432 (8.5)	1638 (9.7)	1933 (11.4)	2055 (12.0)
	aRR (95% CI)*	1 (reference)	1.05 (0.98–1.13)	1.19 (1.11–1.28)	1.40 (1.31–1.50)	1.48 (1.38–1.58)
**Residence before hospital admission**	Total No	1343	1401	1638	1933	2055
Own living environment	No. (%)	1279 (95.2)	1332 (95.1)	1567 (95.7)	1816 (93.9)	1943 (94.5)
(Other) hospital^a^	No. (%)	35 (2.6)	48 (3.4)	41 (2.5)	76 (3.9)	80 (3.9)
Other^b^	No. (%)	29 (2.2)	21 (1.5)	30 (1.8)	41 (2.1)	32 (1.6)
**Destination after hospital discharge**	Total No	1343	1403	1638	1933	2055
Own living environment	No. (%)	1085 (80.8)	1106 (78.8)	1260 (76.9)	1473 (76.2)	1523 (74.1)
	RR (95% CI)	1 (reference)	0.98 (0.90–1.06)	0.95 (0.88–1.03)	0.94 (0.87–1.02)	0.92 (0.85–0.99)
Institutionalization	No. (%)	205 (15.3)	245 (17.5)	312 (19.0)	379 (19.6)	438 (21.3)
	RR (95% CI)	1 (reference)	1.14 (0.95–1.38)	1.25 (1.05–1.49)	1.28 (1.08–1.52)	1.40 (1.18–1.65)
Psychiatric hospital	No. (%)	41 (3.1)	57 (4.1)	66 (4.0)	89 (4.6)	129 (6.3)
	RR (95% CI)	1 (reference)	1.33 (0.89–1.99)	1.32 (0.89–1.95)	1.51 (1.04–2.18)	2.06 (1.45–2.92)
(Other) hospital^c^	No. (%)	40 (3.0)	38 (2.7)	51 (3.1)	75 (3.9)	75 (3.6)
	RR (95% CI)	1 (reference)	0.91 (0.58–1.42)	1.05 (0.69–1.58)	1.30 (0.89–1.91)	1.23 (0.83–1.80)
Other^d^	No. (%)	124 (9.2)	150 (10.7)	195 (11.9)	215 (11.1)	234 (11.4)
	RR (95% CI)	1 (reference)	1.16 (0.91–1.47)	1.29 (1.03–1.61)	1.20 (0.97–1.50)	1.23 (0.99–1.53)
Death	No. (%)	53 (3.9)	52 (3.7)	66 (4.0)	81 (4.2)	94 (4.6)
	RR (95% CI)	1 (reference)	0.94 (0.64–1.38)	1.02 (0.71–1.47)	1.06 (0.75–1.50)	1.16 (0.83–1.62)

aRR, adjusted risk ratio; RR, risk ratio; CI, confidence interval.

*Adjusted for age and sex with direct standardization; 2013 cohort was selected as a reference.

^a^Academic, general, categorical, psychiatric.

^b^Rehabilitation institution, nursing/residential home, other institutions, hospital abroad, born in this hospital, origin unknown.

^c^Academic, general, categorical.

^d^Rehabilitation institution, nursing/residential home, other institutions, hospital abroad, hospice, destination unknown.

Opioid poisoning cases were identified in the hospitalization dataset by the ICD-10CM codes reported in the Supplement online.

## Discussion

We previously reported an increase in opioid prescription and related fatalities in the Netherlands from 2013 to 2017^2^. In the present study, based on national statistics, and annual population-wide national surveys we further elaborated on causes and consequences of the increase in opioid use. We found a shift from NSAID prescription to opioid prescription, without an overall increase in need for pain treatment. Over the four-year period, opioid prescriptions increased by 20%, whereas opioid poising increased by nearly 50%. The increase in opioid prescriptions was mainly due to a large increase in the use of natural opioids. The severity of opioid poisoning also increased, since there were more opioid-related deaths among those admitted with opioid poisoning, and the number of those who were consequently transferred to the other care facilities had risen.

The increase in opioid prescriptions was mainly due to an increase in natural opioid prescriptions (N02AA), namely, morphine, hydromorphone, and especially oxycodone, and not in synthetic opioid prescriptions (N02AZ). At the same time a decrease in NSAIDs prescription rate has been observed, although still a large proportion of residents (13.7% of the total population) received an NSAIDs prescription in 2017. Of note, NSAIDs are not only prescribed, but are also sold over the counter in the Netherlands^26–28^. We demonstrated, that a recent increase in opioid prescription cannot be sufficiently explained by increase in prevalence and severity of pain, but that changed analgesic prescription practice, defined as a shift from NSAIDs to opioids prescription, is the most probable reason for opioid epidemic in the Netherlands. The change in analgesic prescription practice subsequently stemmed from a concurrent introduction of oxycodone by revised Dutch pain treatment guidelines^8^, and restriction of NSAIDs use due to their common adverse events by the scientific community^29, 30^.

We considered suspicion bias in the observed increase of opioid-related fatalities^31^. When suspicion bias would have been the explanation for the increased rate of opioid-related fatalities, an increase in hospital admission and death due to opioid poisoning would have been restricted to those receiving an opioid prescription in the same calendar year. However, the increase also included individuals who had not received opioid prescriptions. These individuals most probably had acquired the drugs illegally, since we found a few in-hospital poisoning (3.9% of all opioid poisonings in 2017). Furthermore, those who had not received an opioid prescription were considerably younger (mean age difference was ten years) compared with those receiving opioid prescriptions and mostly males (65% of all opioid poisoning cases in those not having prescription), which further supports our finding that among those who had not received an opioid prescription, opioid poisoning occurred due to illicit opioid use. These observations render suspicion bias as an explanation for the observed increased rate of opioid poisoning unlikely. Furthermore, this finding is consistent with reports on the opioid epidemic from the United States that showed that widespread opioid use leads to widespread opioid addiction (either prescription or illegal use), with gradual increase in severity of consequences (fatal or non-fatal opioid poisoning)^32^.

Opioid use and opioid overdose deaths are increasing in most countries in the European union^33–37^. However, the situation of pharmacologic pain relief in the Netherlands is somewhat different compared with other European countries. For instance, Danish and British pain guidelines advocate NSAIDs as first line treatment, and are far more stringent in opioid prescription compared to the Dutch pain guideline^38, 39^. Furthermore, a *decrease* in prescription opioid use has been noted since 2016 in the United Kingdom^40^. Specifically, in the United Kingdom and in Denmark, tramadol is the most frequently prescribed opioid, and not natural opioids, such as oxycodone (the opioid that is advocated by Dutch pain guidelines in favour of NSAIDs)^41, 42^. This reinforces our finding that a changed analgesic prescription practice, which was preceded by pain guidelines, is indeed responsible for the recent opioid epidemic in the Netherlands. In addition, we showed that, over time, more patients in the Netherlands suffered from prescription opioid poisoning than from illegal opioid poisoning, while in the United Kingdom the far majority of patients have opioid poisoning related with heroin use, i.e., illegal opioid poisoning^33^, further supporting the evidence that the opioid epidemic in the Netherlands is for a large part iatrogenic.

This research has some methodological issues that warrant commenting. First, to estimate pain prevalence and severity we used results of national health surveys. All other outcomes were identified in the Dutch national statistics. Second, the question related to pain prevalence was missing in 46% of participants. As we consider it unlikely that the missingness was at random, while at the same time the amount of missingness was large, we decided not to impute missing values. However, we consider it unlikely that the reason for missingness changed over the observation period and the level of missingness did not change between 2014 and 2017. Third, non-response bias cannot be excluded as survey response rates ranged between 60 to 65%, depending on the year. Although, this is an acceptable response rate of questionnaires in social sciences^43^, it may have affected our results. However, population characteristics of survey participants were similar to the total Dutch population from 2014 to 2017 (see the Supplementary Table S1 online), as well as participants were randomly sampled. Therefore, it is unlikely that the results of the national survey are not representative of the whole population. Nevertheless, the missing data may render the absolute numbers of individuals reporting pain inaccurate, where an overestimate seems most likely. Additionally, the frequency of opioid and NSAIDs prescription among the respondents of surveys was similar to the frequency of opioid and NSAIDs prescription in the total Dutch population (see the Supplementary Table S4 online). Fourth, we did not have the detailed prescription information to enable us to identify individual active substances, dosing, and pharmaceutical dosage forms. Fifth, we only performed research into opioid and NSAIDs prescriptions, but not into other analgesic agents, such as antidepressants and antiepileptic agents, as those are mostly used for neuropathic pain^44^. Sixth, NSAIDs are also available as an over the counter medication, and the information about the proportion of the population exposed to them is unknown. Although, the NSAIDs prescription decreased between 2013 and 2017 in the Netherlands, that does not necessarily imply that the total exposure to NSAIDs in the total population decreased, because NSAIDs are also available over the counter. Lastly, hospital diagnosis and deaths were ICD-10 coded by the CBS and the accuracy of these codes is not known.

In conclusion, the opioid prescription rate is increasing in the Netherlands, without an increase in pain prevalence and severity. This increase is mainly related to natural opioid use, while at the same time NSAIDs prescription is decreasing. This shift in analgesic prescription practice was accompanied by the increase and the worsening of opioid toxicity, which was related to prescription opioids and increased illicit use of opioids. The changed analgesic pharmacotherapy strategy in the Netherlands has potentially exposed more individuals to toxic effects of opioid use, and without taking any measures to prevent further deterioration of pain management, it doesn’t seem that rate of opioid-related fatalities will decline any time soon.

## Supplementary information

Supplementary information.

## Data Availability

Data obtained in all analyses cannot be shared with third parties as Statistics Netherlands does not permit this.
