# Supplementary material for: Causes and consequences of the opioid epidemic in the Netherlands: a population-based cohort study
Source: Sci Rep. 2020 Sep 17;10:15309. doi: 10.1038/s41598-020-72084-6 (PMC7499208; doi:10.1038/s41598-020-72084-6)
Supplement: Supplementary file 1 — Supplementary information. [file 41598_2020_72084_MOESM1_ESM.pdf]

Supplement to: Causes and consequences of the opioid epidemic in the Netherlands: a population-based cohort study

Authors: Ajda Bedene, MPharm[1,2]; Eveline L.A. van Dorp, MD MSc PhD[2]; Tariq Faquih, MSc[1]; Prof Suzanna C. Cannegieter, MD PhD[1,3]; Dennis O. Mook-Kanamori, MD PhD[1,4]; Marieke Niesters, MD PhD[2]; Monique van Velzen, PhD[2]; Maaike G.J. Gademan, PhD[1,5]; Prof Frits R. Rosendaal, MD PhD[1]; Prof Marcel L. Bouvy, PharmD PhD[6]; Prof Albert Dahan, MD PhD[2]; Willem M. Lijfering, MD PhD[1*]

Content

STROBE Statement—Checklist of items that should be included in reports of cohort studies

International Statistical Classification of Diseases and Related Health Problems, 10th revision of the World Health Organization used to identify opioid-related complications in the Hospital and Mortality data, the Netherlands, from 2013 to 2017

Supplementary Table S1. Characteristics of the study population: total Dutch population and the GE survey cohort, from 2013 to 2017

Supplementary Table S2. Age- and sex- adjusted relative risks and incidence rates for opioid prescription cases, overall and stratified by natural and synthetic, and NSAIDs prescription cases in the Netherlands, from 2013 to 2017

Supplementary Table S3. Age- and sex- adjusted relative risks and incidence rates for opioid prescription vs NSAIDs prescription cases, stratified by concomitant and only prescription, in the Netherlands, from 2013 to 2017

Supplementary Table S4. Opioid and NSAIDs prescription rate among respondents of GE surveys, from 2014 to 2017

STROBE Statement—Checklist of items that should be included in reports of cohort studies

	Item No	Recommendation	Page No
**Title and abstract**	1	(*a*) Indicate the study's design with a commonly used term in the title or the abstract	1
		(*b*) Provide in the abstract an informative and balanced summary of what was done and what was found	3
**Introduction**			
Background/rationale	2	Explain the scientific background and rationale for the investigation being reported	4,5
Objectives	3	State specific objectives, including any prespecified hypotheses	5
**Methods**			
Study design	4	Present key elements of study design early in the paper	6
Setting	5	Describe the setting, locations, and relevant dates, including periods of recruitment, exposure, follow-up, and data collection	6
Participants	6	(*a*) Give the eligibility criteria, and the sources and methods of selection of participants. Describe methods of follow-up	6-10
		(*b*) For matched studies, give matching criteria and number of exposed and unexposed	NA
Variables	7	Clearly define all outcomes, exposures, predictors, potential confounders, and effect modifiers. Give diagnostic criteria, if applicable	6-10
Data sources/ measurement	8*	For each variable of interest, give sources of data and details of methods of assessment (measurement). Describe comparability of assessment methods if there is more than one group	6-10
Bias	9	Describe any efforts to address potential sources of bias	15
Study size	10	Explain how the study size was arrived at	NA
Quantitative variables	11	Explain how quantitative variables were handled in the analyses. If applicable, describe which groupings were chosen and why	6-10
Statistical methods	12	(*a*) Describe all statistical methods, including those used to control for confounding	8,9
		(*b*) Describe any methods used to examine subgroups and interactions	8,9
		(*c*) Explain how missing data were addressed	8,9
		(*d*) If applicable, explain how loss to follow-up was addressed	NA
		(*e*) Describe any sensitivity analyses	NA
**Results**			
Participants	13*	(a) Report numbers of individuals at each stage of study—eg numbers potentially eligible, examined for eligibility, confirmed eligible, included in the study, completing follow-up, and analysed	11
		(b) Give reasons for non-participation at each stage	NA
		(c) Consider use of a flow diagram	NA
Descriptive data	14*	(a) Give characteristics of study participants (eg demographic, clinical, social) and information on exposures and potential confounders	11
		(b) Indicate number of participants with missing data for each variable of interest	11
		(c) Summarise follow-up time (eg, average and total amount)	NA
Outcome data	15*	Report numbers of outcome events or summary measures over time	11-13

Main results	16	(*a*) Give unadjusted estimates and, if applicable, confounder-adjusted estimates and their precision (eg, 95% confidence interval). Make clear which confounders were adjusted for and why they were included	11-13
		(*b*) Report category boundaries when continuous variables were categorized	11-13
		(*c*) If relevant, consider translating estimates of relative risk into absolute risk for a meaningful time period	11-13
Other analyses	17	Report other analyses done—eg analyses of subgroups and interactions, and sensitivity analyses	11-13
**Discussion**			
Key results	18	Summarise key results with reference to study objectives	14
Limitations	19	Discuss limitations of the study, taking into account sources of potential bias or imprecision. Discuss both direction and magnitude of any potential bias	16
Interpretation	20	Give a cautious overall interpretation of results considering objectives, limitations, multiplicity of analyses, results from similar studies, and other relevant evidence	14-17
Generalisability	21	Discuss the generalisability (external validity) of the study results	14-17
**Other information**			
Funding	22	Give the source of funding and the role of the funders for the present study and, if applicable, for the original study on which the present article is based	18

*Give information separately for exposed and unexposed groups.

Note: An Explanation and Elaboration article discusses each checklist item and gives methodological background and published examples of transparent reporting. The STROBE checklist is best used in conjunction with this article (freely available on the Web sites of PLoS Medicine at http://www.plosmedicine.org/, Annals of Internal Medicine at http://www.annals.org/, and Epidemiology at http://www.epidem.com/). Information on the STROBE Initiative is available at http://www.strobe-statement.org.

International Statistical Classification of Diseases and Related Health Problems, 10th revision of the World Health Organization used to identify opioid-related complications in the Hospital and Mortality data, the Netherlands, from 2013 to 2017

ICD-10CM opioid poisoning	
F11.0	Acute intoxication
F11.1	Harmful use
F11.2	Dependence syndrome
F11.3	Withdrawal state
F11.4	Withdrawal state with delirium
F11.5	Psychotic disorder
F11.6	Amnesic syndrome
F11.7	Residual and late-onset psychotic disorder
F11.8	Other mental and behavioural disorders
F11.9	Unspecified mental and behavioural disorder
T40.0	Opium poisoning
T40.1	Heroin poisoning
T40.2	Poisoning by other opioids
T40.3	Methadone poisoning
T40.4	Poisoning by other synthetic narcotics
T40.6	Poisoning by other and unspecified narcotics

Supplementary Table S1. Characteristics of the study population: total Dutch population and the GE survey cohort, from 2013 to 2017

		**2013**	**2014**	**2015**	**2016**	**2017**
**The total Dutch population**	No.	16779575	16829290	16900726	16979120	17081507
	Mean age, years	40.8	41.0	41.3	41.5	41.6
	Male, No. (%)	8307339 (49.5)	8334385 (49.5)	8372858 (49.5)	8417135 (49.6)	8475102 (49.6)
	Female, No. (%)	8472236 (50.5)	8494905 (50.5)	8527868 (50.5)	8561985 (50.4)	8606405 (50.4)
**The GE cohort**	No.	NA	9516	9358	9165	9826
	Mean age (SD), years	NA	40.4 (23.7)	40.7 (23.5)	41.0 (23.7)	41.7 (24.1)
	Male, No. (%)	NA	4637 (48.7)	4609 (49.3)	4455 (48.6)	4848 (49.3)
	Female, No. (%)	NA	4879 (51.3)	4749 (50.7)	4710 (51.4)	4978 (50.7)

Abbreviations: SD, standard deviation; NA, not available

Supplementary Table S2. Age- and sex- adjusted relative risks and incidence rates for opioid prescription cases, overall and stratified by natural and synthetic, and NSAIDs prescription cases in the Netherlands, from 2013 to 2017

		2013 (n=16779575)	2014 (n=16829290)	2015 (n=16900726)	2016 (n=16979120)	2017 (n=17081507)
**Opioid prescription**	No.	814211	863110	921754	975979	1027019
	aIR (per 100,000, 95% CI)*	4850 (4840-4860)	5080 (5070-5090)	5351 (5340-5362)	5597 (5586-5608)	5819 (5808-5831)
	aRR (95% CI)*	1 (reference)	1.05 (1.04-1.05)	1.10 (1.10-1.11)	1.15 (1.15-1.16)	1.20 (1.20-1.20)
Natural	No.	183549	232252	297498	369172	431412
	aIR (per 100,000, 95% CI)*	1090 (1090-1100)	1360 (1360-1370)	1723 (1716-1729)	2111 (2105-2118)	2437 (2430-2444)
	aRR (95% CI)*	1 (reference)	1.25 (1.24-1.25)	1.57 (1.57-1.58)	1.93 (1.92-1.94)	2.23 (2.22-2.24)
Synthetic	No.	630662	630858	624256	606807	595599
	aIR (per 100,000, 95% CI)*	3760 (3750-3770)	3710 (3700-3720)	3628 (3619-3637)	3485 (3477-3494)	3382 (3374-3391)
	aRR (95% CI)*	1 (reference)	0.99 (0.98-0.99)	0.97 (0.96-0.97)	0.93 (0.92-0.93)	0.90 (0.90-0.90)
**NSAIDs prescription**	No.	2600896	2535617	2469770	2405557	2345221
	aIR (per 100,000, 95% CI)*	15500 (15480-15520)	15030 (15010-15040)	14532 (14514-14550)	14050 (14032-14068)	13587 (13570-13604)
	aRR (95% CI)*	1 (reference)	0.97 (0.97-0.97)	0.94 (0.94-0.94)	0.91 (0.90-0.91)	0.88 (0.88-0.88)

Abbreviations: aRR, adjusted risk ratio; CI, confidence interval; aIR, adjusted incidence rate; NSAIDs, nonsteroidal anti-inflammatory drugs
*adjusted for age and sex with direct standardization; the 2013 total Dutch population cohort was selected as a reference.
Individuals, who reimbursed opioid prescriptions were selected by ATC code N02A (natural, N02AA; synthetic N02AZ), NSAIDs prescriptions by ATC code M01A.

Supplementary Table S3. Age- and sex- adjusted relative risks and incidence rates for opioid prescription vs NSAIDs prescription cases, stratified by concomitant and only prescription, in the Netherlands, from 2013 to 2017

Opioid prescription	NSAIDs prescription		2013 (n=16779575)	2014 (n=16829290)	2015 (n=16900726)	2016 (n=16979120)	2017 (n=17081507)
**Yes**	**No**	No.	398029	432597	469045	508907	542168
		aIR (per 100,000, 95% CI)*	2372 (2365-2379)	2535 (2527-2542)	2703 (2695-2711)	2890 (2883-2898)	3036 (3028-3044)
		aRR (95% CI)*	1 (reference)	1.07 (1.06-1.07)	1.14 (1.13-1.14)	1.22 (1.21-1.22)	1.28 (1.27-1.29)
**Yes**	**Yes**	No.	416182	430513	452709	467072	484851
		aIR (per 100,000, 95% CI)*	2480 (2473-2488)	2543 (2535-2550)	2648 (2640-2655)	2706 (2699-2714)	2783 (2775-2791)
		aRR (95% CI)*	1 (reference)	1.03 (1.02-1.03)	1.07 (1.06-1.07)	1.09 (1.09-1.10)	1.12 (1.12-1.13)
**No**	**Yes**	No.	2184714	2105103	2017061	1938485	1860370
		aIR (per 100,000, 95% CI)*	13020 (13003-13037)	12483 (12466-12499)	11884 (11868-11900)	11344 (11328-11360)	10804 (10788-10819)
		aRR (95% CI)*	1 (reference)	0.96 (0.96-0.96)	0.91 (0.91-0.91)	0.87 (0.87-0.87)	0.83 (0.83-0.83)
**No**	**No**	No.	13780650	13861007	13961911	14064656	14194118
		aIR (per 100,000, 95% CI)*	82128 (82084-82171)	82440 (82396-82483)	82765 (82722-82809)	83059 (83016-83103)	83377 (83333-83420)
		aRR (95% CI)*	1 (reference)	1.00 (1.00-1.00)	1.01 (1.01-1.01)	1.01 (1.01-1.01)	1.02 (1.01-1.02)

Abbreviations: aRR, adjusted risk ratio; CI, confidence interval; aIR, adjusted incidence rate; NSAIDs, nonsteroidal anti-inflammatory drugs
*adjusted for age and sex with direct standardization; the 2013 total Dutch population cohort was selected as a reference.
Individuals, who reimbursed opioid prescriptions were selected by ATC code N02A (natural, N02AA; synthetic N02AZ), NSAIDs prescriptions by ATC code M01A.

Supplementary Table S4. Opioid and NSAIDs prescription rate among respondents of GE surveys, from 2014 to 2017

		2014 (n=9516)	2015 (n=9358)	2016 (n=9165)	2017 (n=9826)
**Opioid prescription**	No./ Total No. (%)	447/9516 (4.70)	468/9358 (5.00)	504/9165 (5.50)	576/9826 (5.86)
	aRR (95% CI)*	1 (reference)	1.05 (0.93-1.20)	1.15 (1.02-1.31)	1.19 (1.05-1.34)
**Pain impeded activities of daily living**					
Not at all	No./ Total No. (%)	70/2287 (3.06)	76/2191 (3.47)	75/2117 (3.54)	94/2330 (4.03)
	aRR (95% CI)*	1 (reference)	1.15 (0.83-1.59)	1.17 (0.84-1.62)	1.27 (0.93-1.74)
Somewhat	No./ Total No. (%)	94/1857 (5.06)	112/1861 (6.02)	119/1819 (6.54)	143/1923 (7.44)
	aRR (95% CI)*	1 (reference)	1.17 (0.89-1.54)	1.29 (0.98-1.68)	1.38 (1.06-1.79)
Moderate	No./ Total No. (%)	92/516 (17.83)	74/502 (14.74)	85/511 (16.63)	99/506 (19.57)
	aRR (95% CI)*	1 (reference)	0.81 (0.60-1.10)	0.95 (0.70-1.27)	1.05 (0.79-1.39)
Much and extreme	No./ Total No. (%)	130/459 (28.32)	135/467 (28.91)	159/500 (31.8)	166/498 (33.33)
	aRR (95% CI)*	1 (reference)	1.01 (0.79-1.29)	1.13 (0.89-1.42)	1.18 (0.93-1.48)
Missing	No./ Total No. (%)	60/4397 (1.36)	69/4337 (1.59)	66/4218 (1.56)	74/4569 (1.62)
**NSAIDs prescription**	No./ Total No. (%)	1378/9516 (14.48)	1317/9358 (14.07)	1314/9165 (14.34)	1339/9826 (13.63)
	aRR (95% CI)*	1 (reference)	0.96 (0.89-1.03)	0.98 (0.91-1.06)	0.93 (0.86-1.00)
**Pain impeded activities of daily living**					
Not at all	No./ Total No. (%)	334/2287 (14.60)	278/2191 (12.69)	286/2117 (13.51)	299/2330 (12.83)
	aRR (95% CI)*	1 (reference)	0.87 (0.75-1.03)	0.93 (0.79-1.09)	0.87 (0.75-1.02)
Somewhat	No./ Total No. (%)	377/1857 (20.30)	399/1861 (21.44)	362/1819 (19.9)	398/1923 (20.70)
	aRR (95% CI)*	1 (reference)	1.06 (0.92-1.22)	0.98 (0.85-1.13)	1.00 (0.87-1.15)
Moderate	No./ Total No. (%)	168/516 (32.56)	146/502 (29.08)	141/511 (27.59)	163/506 (32.21)
	aRR (95% CI)*	1 (reference)	0.89 (0.71-1.11)	0.84 (0.67-1.05)	0.96 (0.77-1.19)
Much and extreme	No./ Total No. (%)	173/459 (37.69)	187/467 (40.04)	200/500 (40.00)	182/498 (36.55)
	aRR (95% CI)*	1 (reference)	1.03 (0.84-1.27)	1.04 (0.85-1.28)	0.96 (0.78-1.18)
Missing	No./ Total No. (%)	326/4397 (7.41)	307/4337 (7.08)	324/4218 (7.68)	297/4569 (6.50)

Abbreviations: aRR, adjusted risk ratio; CI, confidence interval; GE, Health Interview Survey; NSAIDs, nonsteroidal anti-inflammatory drugs
*adjusted for age and sex with direct standardization; 2014 cohort was selected as a reference population in the GE survey.
